# The Identification of Small Molecule Inhibitors That Reduce Invasion and Metastasis of Aggressive Cancers

**DOI:** 10.3390/ijms22041688

**Published:** 2021-02-08

**Authors:** Arjanneke F. van de Merbel, Onno van Hooij, Geertje van der Horst, Cindy C. M. van Rijt-van de Westerlo, Maaike H. van der Mark, Henry Cheung, Jan Kroon, Gerald W. Verhaegh, Johan Tijhuis, Antoine Wellink, Peter Maas, Henk Viëtor, Jack A. Schalken, Gabri van der Pluijm

**Affiliations:** 1 Department of Urology, Leiden University Medical Center, 2333 ZA Leiden, The Netherlands; a.f.van_de_merbel@lumc.nl (A.F.v.d.M.); g.van_der_horst@lumc.nl (G.v.d.H.); m.h.van_der_mark@lumc.nl (M.H.v.d.M.); h.cheung@omnigen.nl (H.C.); j.kroon@lumc.nl (J.K.); 2 Department of Urology, Radboud University Medical Center, 6525 GA Nijmegen, The Netherlands; onno.vanhooij@radboudumc.nl (O.v.H.); cindy.vandewesterlo-vanrijt@radboudumc.nl (C.C.M.v.R.-v.d.W.); Gerald.verhaegh@radboudumc.nl (G.W.V.); jack.schalken@radboudumc.nl (J.A.S.); 3 Oncodrone BV, 6525 GA Nijmegen, The Netherlands; antoine.wellink@ru.nl (A.W.); henk.vietor@ffund.nl (H.V.); 4 Department of Endocrinology, Leiden University Medical Center, 2333 ZA Leiden, The Netherlands; 5 Specs, 2712 PB Zoetermeer, The Netherlands; johan.tijhuis@specs.net (J.T.); peter.maas@specs.net (P.M.)

**Keywords:** small molecule inhibitors, prostate cancer, breast cancer, bladder cancer, metastasis, invasiveness, E-cadherin

## Abstract

Transformed epithelial cells can activate programs of epithelial plasticity and switch from a sessile, epithelial phenotype to a motile, mesenchymal phenotype. This process is linked to the acquisition of an invasive phenotype and the formation of distant metastases. The development of compounds that block the acquisition of an invasive phenotype or revert the invasive mesenchymal phenotype into a more differentiated epithelial phenotype represent a promising anticancer strategy. In a high-throughput assay based on E-cadherin (re)induction and the inhibition of tumor cell invasion, 44,475 low molecular weight (LMW) compounds were screened. The screening resulted in the identification of candidate compounds from the PROAM02 class. Selected LMW compounds activated E-cadherin promoter activity and inhibited cancer cell invasion in multiple metastatic human cancer cell lines. The intraperitoneal administration of selected LMW compounds reduced the tumor burden in human prostate and breast cancer in vivo mouse models. Moreover, selected LMW compounds decreased the intra-bone growth of xenografted human prostate cancer cells. This study describes the identification of the PROAM02 class of small molecules that can be exploited to reduce cancer cell invasion and metastases. Further clinical evaluation of selected candidate inhibitors is warranted to address their safety, bioavailability and antitumor efficacy in the management of patients with aggressive cancers.

## 1. Introduction

The formation of metastases represents a major problem in the treatment of solid cancers, and 90% of patients suffering from epithelial cancers will eventually die from metastatic disease [1]. The treatment and management of advanced disease in cancer patients are mainly palliative, and new treatment modalities are urgently needed. 

The progression from primary, localized cancer to advanced metastatic disease is a complex, multistep process. Several mechanisms have been delineated that are involved in the acquisition of an invasive tumor phenotype, including epithelial-to-mesenchymal transition (EMT) [2]. EMT is a physiological process in development, postnatal growth and epithelial homeostasis [1]. Under pathological conditions, oncological EMT is reactivated, resulting in a switch from a sessile, epithelial phenotype to a motile, mesenchymal phenotype [3,4,5,6]. One of the key features of EMT is the loss of the epithelial marker E-cadherin and the upregulation of mesenchymal markers (e.g., vimentin and N-cadherin). E-cadherin is encoded by the *CDH1* gene and functions as a homotypic cell–cell adhesion receptor. E-cadherin plays an important role in maintaining tissue integrity and architecture in epithelial tissues. Under physiological conditions, epithelial cells are polarized and, depending on the type of epithelial tissue, possess tight, gap and adherens junctions. In this way, epithelial cells are tightly connected to each other and the basement membrane [7]. In contrast, mesenchymal cells are highly migratory, unpolarized and spindle-shaped [5]. Loss of the epithelial marker E-cadherin and acquisition of mesenchymal markers (e.g., vimentin, N-cadherin/*CDH2* and fibronectin) are associated with a poor prognosis in different types of solid cancers, including prostate, bladder and breast cancers [8,9,10]. 

In this study, we screened over 44,000 low molecular weight (LMW) compounds from multiple compound libraries for their ability to (re)induce E-cadherin expression and to reduce tumor cell invasion in a panel of aggressive cancer types, including prostate, bladder, breast and pancreatic cancers. We identified multiple candidate LMW compounds that block the acquisition of an invasive phenotype. Based on the acquired in vitro data, a selected number of LMW compounds were studied in vitro and in preclinical human prostate and breast cancer progression and metastasis mouse models. Multiple LMW compounds of the PROAM02 class were identified as candidate pharmacologic agents for the treatment of aggressive cancers. 

## 2. Results

### 2.1. Library Screening Strategy

**Figure 1:**
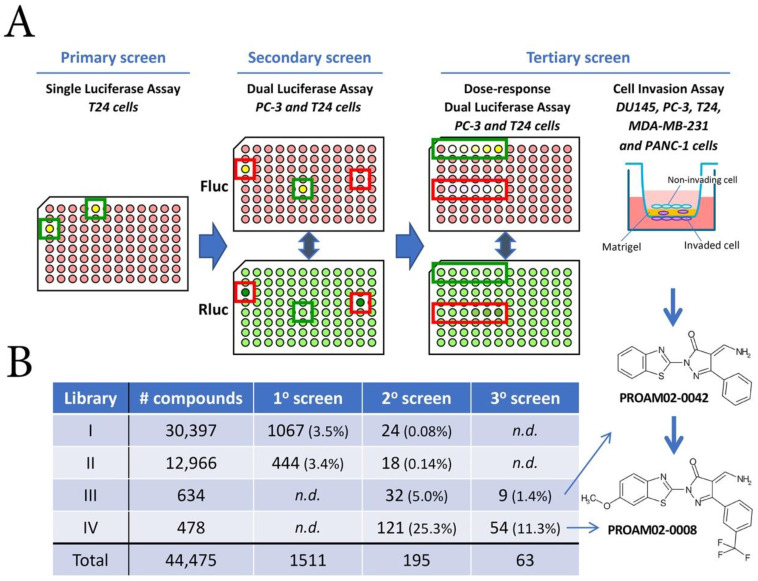
Overview of the screening strategy for the identification of candidate low molecular weight (LMW) compounds (**A**) Three screening rounds were performed with four compound libraries. Initially, libraries I and II were tested in E-cadherin-negative human bladder cancer T24 cells that express the *Ecad-Fluc/Rluc* reporter construct. In the secondary screen, a dual luciferase assay was performed in PC-3 (E-cadherin intermediate-positive) and T24 (E-cadherin-negative) cells that express the *Ecad-Fluc/Rluc* reporter construct. Compounds identified in the primary screening round and compounds from libraries III and IV were used. During the third round of screening, dose-response dual luciferase assays were performed in both reporter models. In addition, invasion assays were performed in multiple human cancer cell lines, including DU145, PC-3, T24, MDA-MB-231 and Panc-1 cells. *Firefly* luciferase-positive wells are indicated in yellow, and *Renilla* luciferase-positive wells are depicted in green. Excluded wells are depicted in red boxes due to aberrant *Renilla* luciferase values. (**B**) Overview of the number of compounds tested and identified in the three screening rounds. N.D. = not determined.

For the first screening round, LMW compound libraries I and II (30,976 and 12,966 LMW compounds) were tested on the established *T24/pEcad-luc/Rluc* E-cadherin reporter (Figure S1) [11]. The first screening round resulted in the identification of 1511 positive compounds (cut-off value of 2.0-fold induction vs. vehicle) (Figure 1A,B). In the second round with *T24/pEcad-luc/Rluc* and *PC-3/pEcad-luc/Rluc* E-cadherin reporter models, 1511 compounds from the first screening were tested, supplemented with libraries III and IV (634 and 478 compounds). This screening round yielded 195 compounds that increased E-cadherin promoter activity (Figure 1B). After dose-response reporter assays and cell invasion assays in the third screening round, 63 compounds were selected that both increased E-cadherin reporter activity and decreased tumor cell invasion capacity dose-dependently in multiple invasive cell lines. One class of compounds, i.e., aminomethylene pyrazolones (WO2013/13193 A1), was selected for the lead optimization, resulting in the identification of the class of PROAM02 compounds.

### 2.2. Selected LMW Compounds Block Tumor Invasion and Aggressiveness In Vitro 

**Figure 2:**
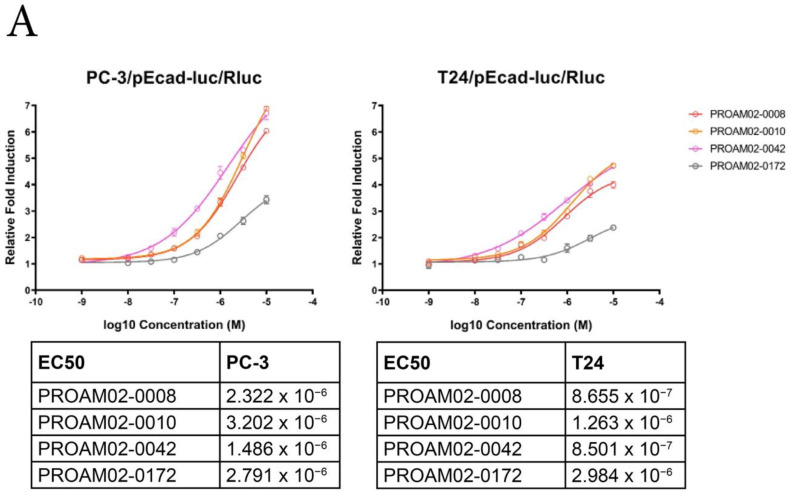

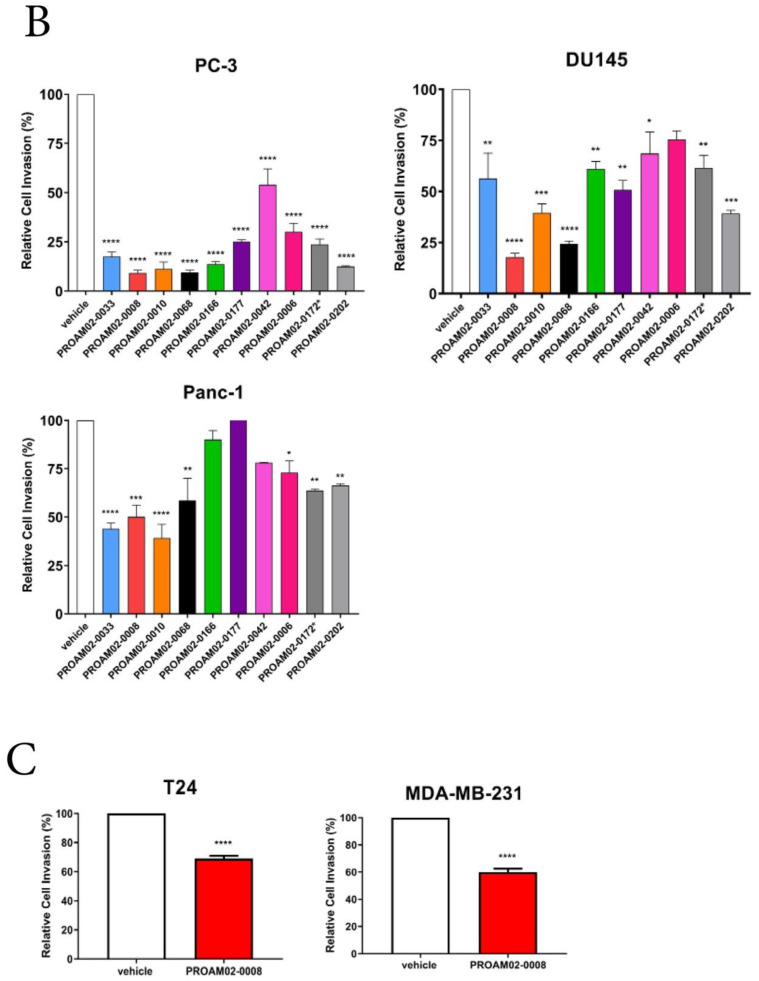
LMW compounds induce E-cadherin reporter activity and reduce invasion in vitro. (**A**) PC-3 and T24 cells were treated with a dose range of LMW compounds. E-cadherin luciferase reporter induction was measured, and EC_50_ values were calculated. (**B**) The effect of LWM compounds (10 µM) on cell invasion in PC-3, DU145 and Panc-1 cells. (**C**) Invasion assays in T24 and MDA-MB-231 cells after treatment with 10-µM PROAM02-0008 * *p* < 0.05, ** *p <* 0.01, *** *p* < 0.001 and **** *p* < 0.0001 (*N* = 2, mean ± SEM, one-way ANOVA and *t*-test).

To study the effect of LMW compounds on the phenotype of invasive potential of cells in vitro, luciferase assays based on E-cadherin reinduction and invasion were performed (Figure 1A and Figures S1 and S2A,B). Several LMW compounds induced a dose-dependent increase in E-cadherin expression, as assessed by luciferase assays and mRNA expression in human cancer cell lines (Figure 2A and Figure S1C). LMW compounds that were able to reinduce E-cadherin expression were analyzed for their ability to block Matrigel invasion in multiple human cancer cell lines. Treatment with selected LMW compounds significantly inhibited cell migration and invasion in invasive cancer cell lines: PC-3, DU145 and Panc-1 (Figure 2B and Figure S2). Most selected LMW compounds displayed a dose-dependent inhibition of invasiveness in human prostate cancer PC-3 cells (Figure S2B). 

PROAM02-0008 was identified as a promising candidate agent, showing an even stronger inhibition of PC-3 cell invasion up to 90% at 10 µM compared to the initial lead compound PROAM02-0042 in PC-3 cells (Figure 2B). Similar to the results in PC-3, DU145 and Panc-1 cell lines, treatment with 10-µM PROAM02-0008 resulted in a significant inhibitory effect on invasion in the MDA-MB-231 and T24 cells (*p* < 0.0001) (Figure 2C). Based on the ability to induce E-cadherin (re)expression and inhibit migration and invasion in various cancer cell lines, the PROAM02 class of chemical compounds and—more specifically—PROAM02-0008 were identified as candidate drugs. 

The selected LMW compounds, including PROAM02-0008, did not affect the proliferation of human prostate and bladder cancer cells in vitro (Figure S3A). However, selected LMW compounds induced cytoskeletal actin rearrangements in invasive prostate and bladder cancer cells, indicative of a less invasive and more differentiated, epithelial phenotype (Figure S3B). 

The acquisition of an invasive phenotype has been previously linked to cancer stem/progenitor cell properties [12,13]. For this, human prostate and bladder cancer cells were evaluated in clonogenic assays in the presence of LMW compounds. Five-micrometer LMW compounds had a significant inhibitory effect on the clonogenic potential (Figure S3C).

### 2.3. Selected LMW Compounds Reduce the Expression of EMT Transcription Factors, Mesenchymal Markers and Antagonize TGF-β Signaling

**Figure 3:**
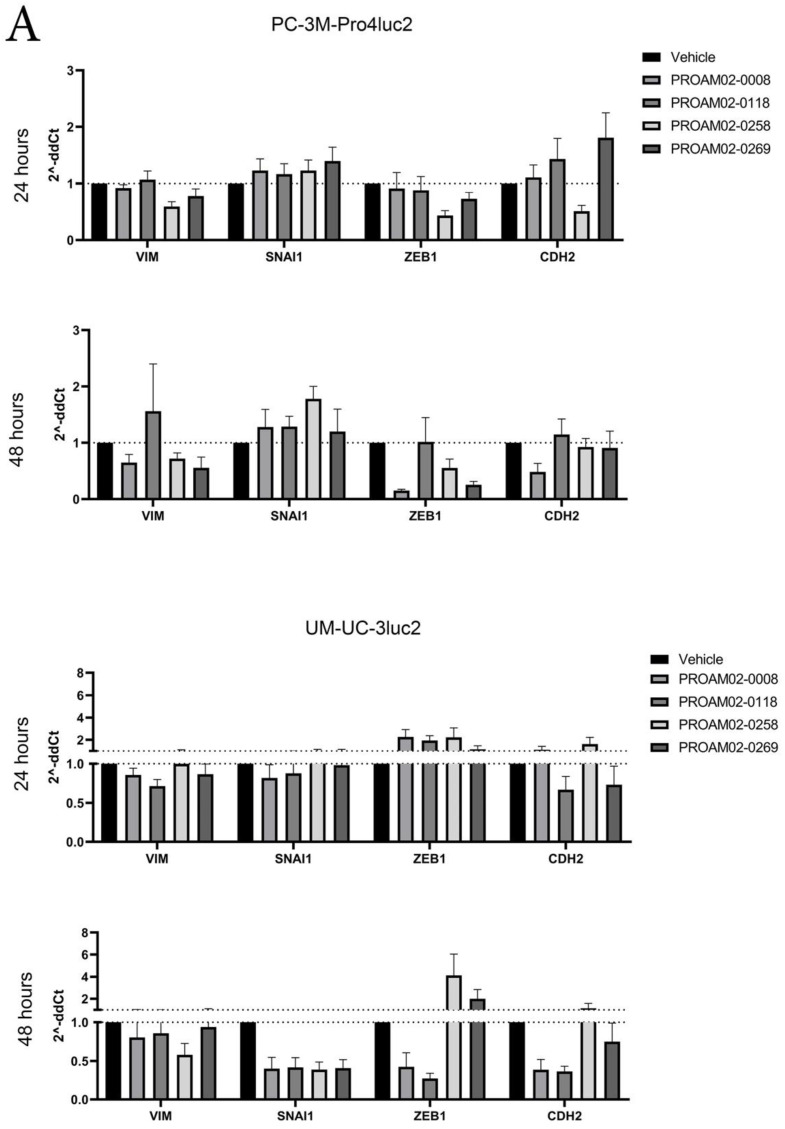

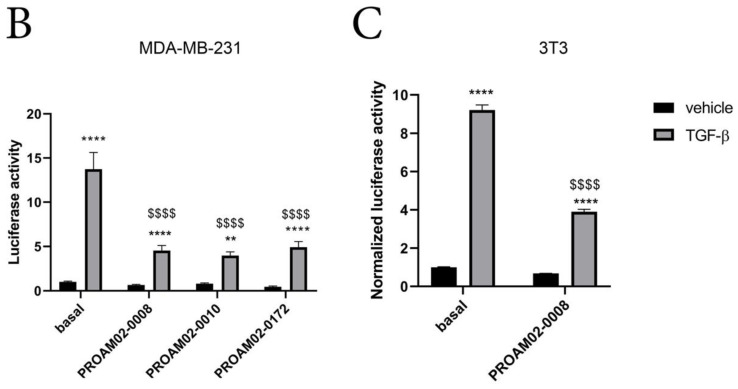
LMW compounds reduce the mRNA expression of mesenchymal markers and decrease Smad2/3-dependent TGF-β reporter activity. (**A**) PC-3M-Pro4luc2 and UM-UC-3luc2 cells were stimulated with 5-µM LMW compounds, and mRNA expression was assessed after 24 and 48 h. The LMW compounds reduced the expression of vimentin, N-cadherin, SNAI1 and ZEB1 in a time-dependent manner. (**B**) The effect of LMW compound PROAM02-0008 alone or combined with TGF-β stimulation on the Smad2/3-dependent CAGA-*Firefly* luciferase reporter activity in human breast cancer cells MDA-MB-231. (**C**) The effect of PROAM02-0008, alone or in combination with TGF-β, on the stimulation of CAGA12-*Firefly* luciferase reporter activity in murine 3T3 fibroblasts. Asterisks indicate vehicle vs. TGF-β1 treatment, and dollar signs indicate basal TGF-β compared to compound + TGF-β1 treatment). ** *p* < 0.01, **** *p* < 0.0001 and $$$$ *p* < 0.0001 (*N* = 2, mean ± SEM, two-way ANOVA).

The acquisition of invasive behavior has been linked to the reduced expression of mesenchymal genes and EMT transcription factors. Treatment with LMW compounds reduced the mRNA expression of mesenchymal markers vimentin and N-cadherin (CDH2) in PC-3M-Pro4luc2 and UM-UC-3luc2 cells after 24 and 48 h. Moreover, the expression of ZEB1 and SNAI1 were reduced upon treatment with the LMW compound (Figure 3A). 

TGF-β represents a key cytokine involved in invasiveness via the induction of ZEB1 and SNAI1 EMT transcription factors [13,14]. For this, we evaluated the effect of selected LMW compounds on SMAD2/3-mediated TGF-β signaling activity in the stable reporter cell line MDA-MB-231/CAGAluc2 [14]. The addition of TGF-β1 resulted in a significant induction of reporter activity compared to vehicle-treated cells (**** *p* < 0.0001) (Figure 3B). In the presence of TGF-β1, PROAM02-0008, -0010 and -0172 significantly antagonized TGF-β-mediated reporter activity ($$$$ *p* < 0.001). The inhibitory effects of PROAM02-0008 on TGF-β signaling was also confirmed in another reporter assay using NIH-3T3 cells that were transfected with the CAGA12 luciferase reporter [15] (Figure 3C). 

### 2.4. Selected LMW Compounds Reduce Tumor Progression and Metastasis in Preclinical In Vivo Models

**Figure 4:**
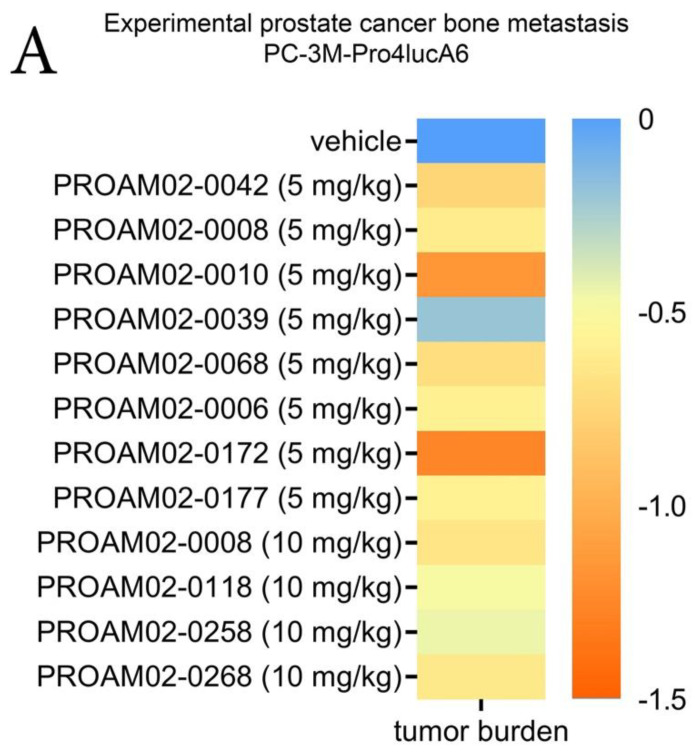

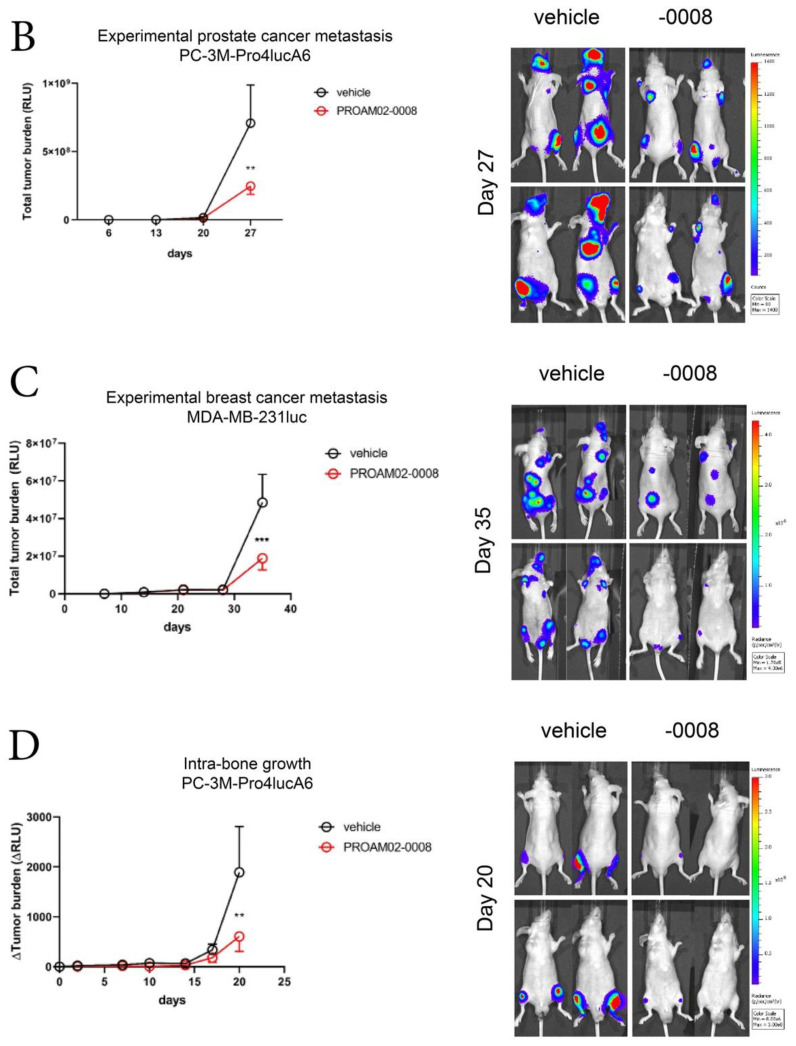
LMW compounds reduce tumor progression and experimental bone metastasis in vivo. (**A**) The effects of daily treatment with the LMW compounds on skeletal metastasis formation in male nude mice after intracardiac inoculation of human prostate cancer cells PC-3M-Pro4 that stably expresses Firefly luciferase. Total tumor burden was assessed by whole-body optical imaging. Total acquired data was log-transformed and normalized to mice receiving vehicle treatment. (**B**) The effect of 5-mg/kg/day PROAM02-0008 on the formation of prostate cancer metastasis in vivo. (**C**) The preventive effect of 5-mg/kg/day PROAM02-0008 on bone metastasis by MDA-MB-231 cells that stably and constitutively express Firefly luciferase. (**D**) The curative effect of 5-mg/kg PROAM02-0008 on the intraosseous growth of human prostate cancer cells. Representative BLI images of mice are shown. Mean tumor burden ± SEM, two-way ANOVA. ** *p* < 0.01 and *** *p* < 0.001.

Next, we assessed whether the selected LMW compounds can inhibit tumor growth and the formation of bone metastases in vivo [12,16]. For this, human prostate cancer cells PC-3M-Pro4luc were inoculated in the left cardiac ventricle of five-week-old male Balb-c nu/nu mice, a well-established model of experimental skeletal metastasis [13,14]. Daily intraperitoneal treatment with LMW compounds resulted in a reduced total tumor burden and number of metastases in vivo compared to vehicle-treated mice (Figure 4A and Figure S4A,B). Treatment with LMW compounds did not affect the body weight of the animals, indicating that the compounds were well-tolerated (Figure S5A,B). 

Based on our in vitro and in vivo data, PROAM02-0008 was selected and evaluated in validated preclinical disease models of experimental metastasis formation and intraosseous growth in vivo [12,13]. Treatment with PROAM02-0008 resulted in a significant reduction of total tumor burden in experimentally induced bone metastasis models using real-time whole-body optical imaging of firefly luciferase-expressing human prostate and bladder cancer cells (Figure 4B,C) (*p* = 0.0073 and *p* = 0.0002). Moreover, treatment with compound PROAM02-0008 significantly reduced the intraosseous growth of human prostate cancer cells (*p* = 0.0049) (Figure 4D). Overall, these results indicate that LMW compounds can inhibit tumor aggressiveness and metastatic behavior in human prostate and breast cancers in vivo.

## 3. Discussion

Epithelial plasticity of cancer cells is involved in the regulation of an invasive phenotype [4,6]. Oncologic EMT is a common process involved in tumor progression and metastasis in different epithelial cancers, including prostate, bladder, breast, pancreatic and liver cancers [17,18,19,20,21,22,23,24,25,26]. Furthermore, oncologic EMT has been linked to the acquisition of therapy resistance, cancer stemness properties and a poor prognosis in cancer patients [4,5,17,27,28]. One of the hallmarks of EMT is the reduced expression of epithelial marker E-cadherin.

In this study, multiple compound libraries of over 44,000 LMW compounds were screened for their ability to (re)induce E-cadherin expression and reduce the invasiveness of a panel of invasive human cancer cell lines. Our study revealed that the class of PROAM02 compounds inhibited cancer cell migration and invasion, clonogenicity and induced E-cadherin re-expression. Furthermore, the administration of selected LMW compounds decreased tumor progression, metastasis formation and intra-bone tumor growth in prostate and breast cancer disease models in vivo. 

The primary read-out for selecting compounds in this study was based on the reinduction of E-cadherin. In addition, we observed that the LMW compounds concomitantly reduced the expression of mesenchymal markers vimentin and N-cadherin, decreased the clonogenic potential of aggressive cancer cells and induced changes in the actin cytoskeleton. Overall, these observations are indicative of differentiation towards a more sessile, epithelial phenotype. One of the factors that can drive tumor invasiveness is TGF-β. TGF-β-induced EMT is mediated—at least in part—by the increased expression of *SNAIs*, *TWIST* and *ZEB* transcription factors that bind E-box sequences of the E-cadherin promoter, thus downregulating its expression. Therefore, interference with TGF-β signaling represents an interesting anticancer approach. In this study, we observed a reduced expression of *ZEB1* and *SNAI1* upon stimulation with the LMW compounds. In addition, we found that the candidate LMW compound PROAM02-0008 induced a significant inhibition of SMAD-dependent TGF-β signaling in two independent reporter assays. These findings support the notion that the tested LMW compounds could interfere with the TGF-β pathway. 

Multiple strategies have been exploited to therapeutically target invasive behavior—for instance, by the targeting of EMT transcription factors [29,30], the translational targeting of the EMT pathway via microRNAs (miRNAs) [31,32] or interference with the EMT pathway by small molecules [33,34]. The LMW compounds identified in this study could be interesting therapeutic options for the treatment of aggressive and invasive epithelial tumors. Our animal studies revealed that the LMW compounds are capable of reducing tumor cell metastasis and intra-bone growth in vivo. This suggests that the PROAM02 class of compounds might be beneficial in both early stage, high-risk organ-confined tumors and in later stages of metastatic disease. However, for the clinical application of these compounds, additional safety, toxicity and efficacy studies are required, and the optimal routes of administration have to be identified. 

## 4. Materials and Methods

### 4.1. Library Screening Strategy

Bladder cancer cells (T24 and UM-UC-3luc2); breast cancer cells (MDA-MB-231, MDA-MB-231luc and MDA-MB-231/CAGAluc2); pancreatic cancer cells (PANC-1); prostate cancer cells (DU145, PC-3, PC-3M-Pro4luc and PC-3M-Pro4luc2); E-cadherin reporter lines T24-*pEcad-luc/Rluc* and PC-3-*pEcad-luc/Rluc* and murine fibroblasts (3T3) were cultured as described in Table S1. Cell lines were propagated for no more than 6 months or 30 passages after resuscitation from stocks. Cell lines were frequently tested for *Mycoplasma* infection, using a *Mycoplasma* specific PCR. Cell lines were authenticated using the PowerPlex 21 System (Promega, Madison, WI, USA) by Eurofins Genomics (Hamburg, Germany).

### 4.2. Reporter Construct

In order to establish a platform for high-throughput screening (HTS) of LMW compound libraries, the *pEcad-Fluc/Rluc* reporter construct was used to generate stable human bladder cancer cell line T24 (E-cadherin-negative) and human prostate cancer cell line PC-3 (E-cadherin intermediate-positive) [11]. In this reporter construct, the *CDH1* (E-cadherin) gene promoter was fused in front of the *Photinus pyralis* luciferase gene (*Fluc*). A constitutively active *Renilla* luciferase reporter (*Rluc*) was used as an internal control (Table S1 and Figure S1). FuGENE-6 transfection reagent was used for the transfections according to manufacturer’s protocols (Promega). 

### 4.3. Screening Strategy of Compound Libraries 

The compound library was provided by Specs (Zoetermeer, The Netherlands). The library was composed of approximately 44,475 small molecular weight compounds and was stored in dimethyl sulfoxide (DMSO) at 10 mM per compound in 96-well plates at −20 °C. Four LMW compound libraries were selected from the Specs screening library for their drug-like properties and their diversity among each other. In the primary screening round, libraries I and II were tested by performing single luciferase assays in T24/*pEcad-luc/Rluc*. Next, a secondary screen was executed by performing dual luciferase assays on T24/*pEcad-luc/Rluc* and PC-3/*pEcad-luc/Rluc*. During the third screening round, libraries III and IV were tested by performing dose-response luciferase assays on T24/*pEcad-luc/Rluc* and PC-3/*pEcad-luc/Rluc* cells and invasion assays on DU145, PC-3, T24, MDA-MB-231 and Panc-1 cells. 

### 4.4. Luciferase Reporter Assays

Two-thousand five hundred T24/*pEcad-luc/Rluc* (clone 1.c.4) or PC-3/*pEcad-luc/Rluc* (clone 2.b.4) cells were seeded in a 96-well culture plate. Three days later, growth medium was replaced by LMW compound-containing growth medium in a final concentration ranging from 1.0 nM to 10 µM or vehicle (≤0.1% DMSO)-containing medium. After 16 h of treatment, the medium was removed, and the cells were rinsed once with PBS. Single and dual luciferase reporter assays were performed, according to the manufacturer’s instructions (Promega), and luciferase activity was measured on a Victor^3^ Multilabel Counter (PerkinElmer, Waltham, MA, USA). In dual luciferase assays, *Firefly* luciferase activity was normalized to *Renilla* luciferase activity. The fold induction in luciferase activity was calculated relative to vehicle (DMSO)-treated cells. Data were analyzed with GraphPad Prism 8 software to determine EC_50_ values of the compounds using a nonlinear fit sigmoidal dose-response curve. 

### 4.5. Cell Invasion Assays

Invasion assays were performed using BioCoat Matrigel Invasion Chambers (Corning, Corning, New York, NY, USA), according to the manufacturer’s instructions. Prior to the invasion assay, cells were incubated in the presence of a compound (10 µM) for four days. Pretreated cells (40,000) were seeded into the invasion chambers in serum-free medium. Compound or vehicle (DMSO) was added to the cells in the upper chamber. The invasion chambers were placed in a 24-well plate containing 500-µL medium with 10% fetal calf serum as a chemo-attractant. As a growth control, the same number of cells was seeded onto the surface of a 24-well culture plate. After 48 h of incubation, noninvading cells remaining above the insert were removed by aspiration and cleaning with cotton swabs. To quantify the total amount of invaded cells, the invasion chamber was then put into CellTiter-Glo Luminescent Cell Viability Reagent (Promega, Madison, WI, USA) and mixed for two minutes on a plate shaker. Luminescent activity was measured on a Victor^3^ Multilabel Counter (PerkinElmer, Waltham, MA, USA). The percentage of cell invasion was calculated as the luminescence on the lower part of the membrane divided by the total luminescence of cells grown on the surface of a 24-well culture plate. Inhibition of cell invasion by a specific compound was calculated relative to vehicle (DMSO)-treated cells.

### 4.6. Clonogenic Assays

In order to investigate the effect of the compounds on the clonogenic capacity, 100 cells were seeded per well in a 6-well plate. After 24 h, the cells were treated with 5 µM of the compounds. After 15–20 days, colonies were fixed with 4% paraformaldehyde (PFA) and subsequently stained with crystal violet. The percentage area covered by colonies was measured by using a colony plugin tool for ImageJ, and the number of colonies was counted. 

### 4.7. Migration and Proliferation Assays

Migration and proliferation assays were performed as previously described [35]. For proliferation assays, 1500 cells were seeded in a 96-well plate, and 24 h later, the cells were treated with 5 μM of the compounds. After 72 h, 20 μL of 3-(4,5 dimethylthiazol-2-yl)-5-(3-carboxymethoxyphenyl)-2-(4-sulfophenyl)-2H-tetrazolium (MTT) (Promega, Madison, WI, USA) was added to each well. After two hours of incubation, mitochondrial activity was measured at 490 nm on a SpectraMax plate reader. 

For the migration assay, cells were starved in medium supplemented with 0.3% serum (starvation medium) overnight. The next day, 60,000 cells were seeded in the presence of a LMW compound in the upper compartment of a Transwell chamber (8 µm, Corning, Corning, New York, NY, USA) in 200-µL starvation medium. Three hundred microliters of medium were added to the lower compartment of the Transwell chamber. After 24 h, the migration assay was stopped, cells were stained with 0.1% crystal violet (Sigma, Saint Louis, MO, USA) and migrated cells were counted.

### 4.8. Phalloidin Staining

A total of 100,000 cells were seeded in 8-well glass chamber slides (Nunc Lab-Tek, Thermo Fisher Scientific, Waltham, MA, USA). After 24 h, cells were treated with 5-µM compounds. After 2 days, cells were fixed with 4% PFA and stained with 0.25-μmol/L phalloidin (Life Technologies, Carlsbad, CA, USA). DAPI was used for nuclear visualization. Representative images were made at 63× magnification (Leica SP8 confocal, Wetzlar, Germany).

### 4.9. RT-qPCR

Human prostate and bladder cancer cells PC-3M-Pro4luc2 and UM-UC-3luc2 were stimulated with 5-μM LMW compound for 24 or 48 h. Subsequently, total RNA was isolated with TRIzol (Invitrogen). cDNA was generated by using random primers (Promega, Madison, WI, USA), and qRT-PCR was performed by using SYBR Green (Bio-Rad, Hercules, CA, USA). Expression was normalized to GAPDH. Primer sequences can be found in Table S2.

### 4.10. TGF-β Dependent Reporter Assays

A stable reporter breast cancer cell line MDA-MB-231/CAGAluc2 was treated with 5-µM LMW compound and 0.1 ng/mL of TGF-β1 (R&D Systems, Minneapolis, MN, USA). After 24 h, luciferase activity was measured [14].

NIH-3T3 cells were transfected with the CAGA12 luciferase [36] and stimulated overnight with 10-µM PROAM02-0008 in the presence or absence of 1.0 ng/mL of TGF-β1 (R&D). Luciferase activity was measured according to the manufacture’s protocols (Bright-Glo Luciferase Assay, Promega, Madison, WI, USA).

### 4.11. In Vivo Experiments

Animal experiments were approved by the local committee for animal welfare of Leiden University (DEC) or the Dutch Central Committee on animal experiments (CCD) and carried out in accordance with the European Communities Council Directive 86/609/EEC (CCD_AVD116002016617, DEC_12127 and DEC_13051).

#### 4.11.1. Preclinical Experimental Metastasis Model

*Firefly* luciferase-expressing PC-3M-Pro4luc or MDA-MB-231luc cells (1 × 10^5^) in 100-µL PBS were inoculated into the left cardiac ventricle of five-week-old male (PC-3M-Pro4luc) or female (MDA-MB-231luc) immunodeficient BALB/c nu/nu mice (RRID:MGI:2160479) (*n* = 15 per group) [13,15]. Prior to inoculation of the cells and to all imaging procedures, mice were anesthetized by isoflurane. One day after inoculation, the mice were attributed to equal experimental groups based on body weight. The mice were treated by daily intraperitoneal administration with either 5 or 10 mg/kg/day of LMW compound or vehicle solution. Tumor progression and metastasis was monitored weekly by whole-body bioluminescent imaging (BLI) after intraperitoneal administration of 50-µL d-luciferin [13,15]. All the imaging was performed using the IVIS Lumina III (Caliper LifeSciences). Tumor burden was defined as relative light units (RLU). The number of metastases was defined after scoring the number of separate bioluminescent foci.

#### 4.11.2. Preclinical Intraosseous Tumor Growth Model

To investigate the curative effect of LMW compounds on intraosseous growth, a single cell suspension of 2.5 × 10^5^ *firefly* luciferase-expressing PC-3M-Pro4luc2 cells in 10-µL PBS was injected into the right tibiae in male 6-8-week-old BALB/c nu/nu (RRID:MGI:2160479) mice, as previously described (*n* = 10 per group) [16]. After 11 days, the mice were daily treated by intraperitoneal injection with 5 or 10 mg/kg of LMW compound or vehicle. Tumor progression was monitored weekly by performing BLI, as described above.

### 4.12. Statistical Analysis

Statistical analysis was performed with GraphPad Prism 8.0 (San Diego, CA, USA). Data is presented as mean ± SEM.

## 5. Conclusions

In conclusion, this study described the identification of selected PROAM02 LMW compounds as promising agents for the treatment of different types of aggressive epithelial cancers. Candidate molecules of the PROAM02 class of LMW compounds may represent an interesting strategy for the treatment of invasive tumors by inducing epithelial differentiation. More studies are warranted to determine the exact translational value and clinical feasibility of the selected candidate pharmacologic agents. 

## 6. Patents

Onno van Hooij, Johan Tijhuis, Henk Viëtor and Jack A. Schalken are inventors on a patent on aminomethylene pyrazolones (WO2013/13193 A1).

## Data Availability

The data presented in this study are available upon reasonable request from the corresponding author.

## Supplementary Materials

The following are available online at https://www.mdpi.com/1422-0067/22/4/1688/s1: Table S1: Overview of cell lines and cell culture conditions. Table S2: RT-qPCR primer sequences. Figure S1: Schematic presentation of the double-reporter construct pEcad-luc/Rluc for the high-throughput screening (HTS) assay. Figure S2: The dose-dependent effect of the LMW compounds on prostate cancer invasion in vitro. Figure S3: Effects of LMW compounds on cancer cell proliferation, organization of the actin cytoskeleton and clonogenicity. Figure S4: The effect of LMW compounds on bone metastasis formation and intraosseous tumor growth in vivo. Figure S5: LMW compound administration does not affect the body weight.
