# Supplementary material for: The Identification of Small Molecule Inhibitors That Reduce Invasion and Metastasis of Aggressive Cancers
Source: Int J Mol Sci. 2021 Feb 8;22(4):1688. doi: 10.3390/ijms22041688 (PMC7915539; doi:10.3390/ijms22041688)
Supplement: Supplementary file 1 [file ijms-22-01688-s001.pdf]

Supplementary Materials

Table S1 Overview of cell lines and cell culture conditions

Cell line		Medium	Supplier	Supplements
DU145	ATCC Cat# HTB-81, RRID:CVCL_0105	RPMI 1640	Lonza, BE12-167F	10% FBS, 100 units/ml penicillin, 50 µg/ml streptomycin, GlutaMAX
PC-3	ATCC Cat# CRL-1435, RRID:CVCL_0035	RPMI 1640	Lonza, BE12-167F	10% FBS, 100 units/ml penicillin, 50 µg/ml streptomycin, GlutaMAX
MDA-MB-231	ATCC Cat# CRM-HTB-26, RRID:CVCL_0062	RPMI 1640	Lonza, BE12-167F	10% FBS, 100 units/ml penicillin, 50 µg/ml streptomycin, GlutaMAX
MDA-MB-231luc		RPMI 1640	Lonza, BE12-167F	10% FBS, 100 units/ml penicillin, 50 µg/ml streptomycin, GlutaMAX, 800 µg/mL of G-418
MDA-MB-231/CAGAluc2		RPMI 1640	Lonza, BE12-167F	0% FBS, 100 units/ml penicillin, 50 µg/ml streptomycin, GlutaMAX, 800 µg/mL of G-418, 0.35 mg/ml hygromycin (Invitrogen)
PANC-1	ATCC Cat# CRL-1469, RRID:CVCL_0480	RPMI 1640	Lonza, BE12-167F	10% FBS, 100 units/ml penicillin, 50 µg/ml streptomycin, GlutaMAX
T24	ATCC Cat# HTB-4, RRID:CVCL_0554	RPMI 1640	Lonza, BE12-167F	10% FBS, 100 units/ml penicillin, 50 µg/ml streptomycin, GlutaMAX

PC-3M-Pro4luc2		Dulbecco’s Modified Eagle medium (DMEM)	Life technologies, Gibco, 31966-021	10% FCII (Hyclone), 100 units/ml penicillin, 50 μg/ml streptomycin<br>800 μg/mL of G-418
PC-3M-Pro4luc (a.k.a. PC-3M-Pro4lucA6)		Dulbecco’s Modified Eagle medium (DMEM)	Life technologies, Gibco, 31966-021	10% FCII (Hyclone), 100 units/ml penicillin, 50 μg/ml streptomycin<br>800 μg/mL of G-418
UM-UC3luc2	ATCC Cat# CRL-1749, RRID:CVCL_1783	Eagle's minimal essential medium (EMEM)	ATCC, 30-2003	10% FBS, 100 units/ml penicillin, 50 μg/ml streptomycin<br>800 μg/mL of G-418
T24-pEcad-luc/Rluc		RPMI 1640	Life Technologies Gibco 31870-025	10% FCS, L-Glutamine, Zeocin (50 μg/ml)
PC-3-pEcad-luc/Rluc		RPMI 1640	Life Technologies Gibco 31870-025	10% FCS, L-Glutamine, Zeocin (50 μg/ml)
3T3	ATCC Cat# CRL-1658, RRID:CVCL_0594	Dulbecco’s Modified Eagle medium (DMEM)	Life technologies, Gibco, 31966-021	10% FCS

Table S2 RT-qPCR primer sequences

GENE	Forward Primer	Reverse Primer
E-cadherin	TTGACGCCGAGAGCTACAC	GACCGGTGCAATCTTCAAA
N-cadherin	CAGACCGACCCAAACAGCAAC	GCAGCAACAGTAAGGACAAACATC
Vimentin	CCAAACTTTTCCTCCCTGAACC	CGTGATGCTGAGAAGTTTCGTTGA
ZEB1	CCATATTGAGCTGTTGCCGC	GCCCTTCCTTTCCTGTGTCA
SNAIL1	ACCACTATGCCGCGCTCTT	GGTCGTAGGGCTGCTGGAA
GAPDH	GACAGTCAGCCGCATCTTC	GCAACAATATCCACTTTACCAGAG

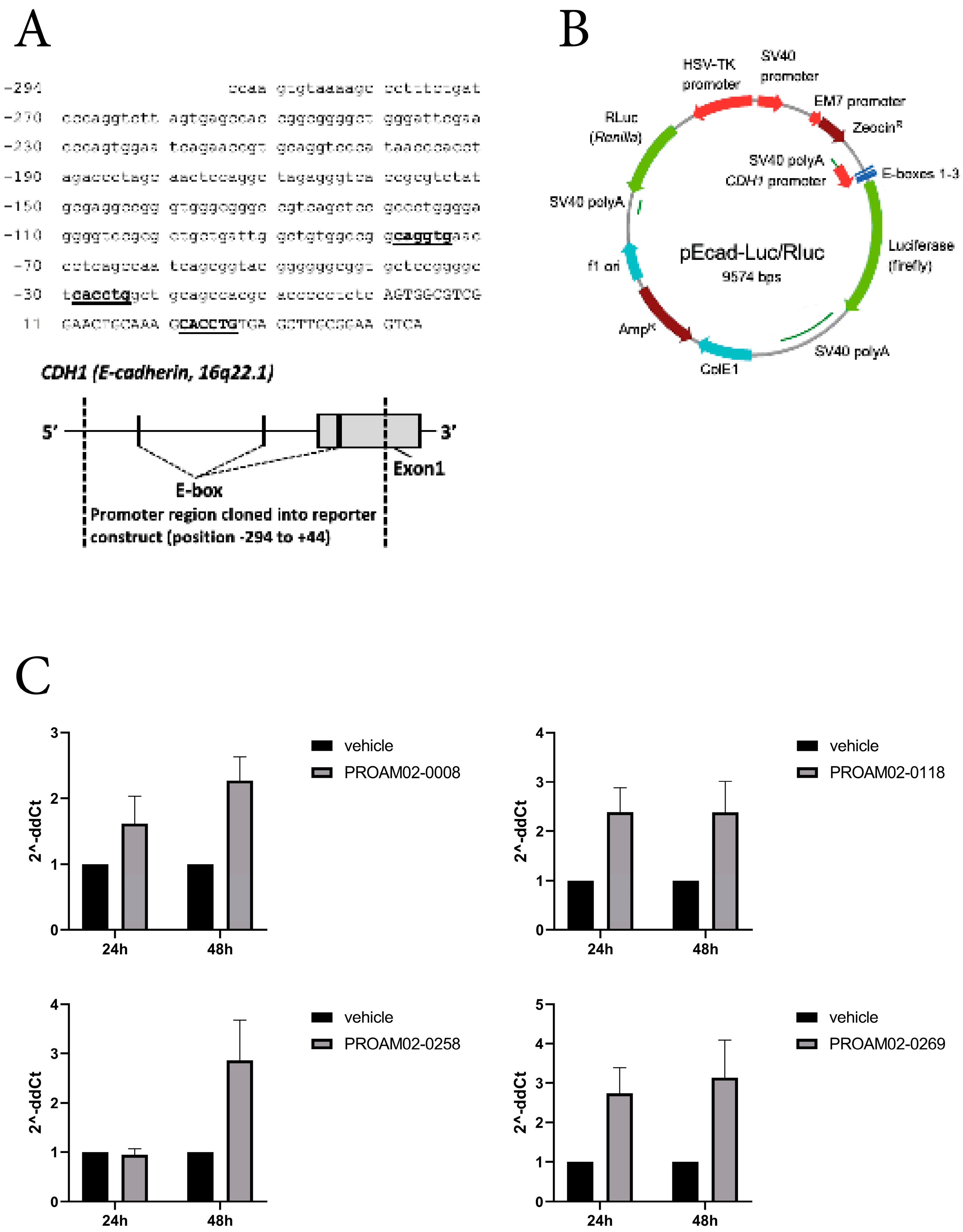

A
CDH1 (E-cadherin, 16q22.1)
5'
3'
E-box
Exon1
Promoter region cloned into reporter construct (position -294 to +44)
B
HSV-TK promoter
SV40 promoter
EM7 promoter
Zeocin^R
RLuc (Renilla)
SV40 polyA
CDH1 promoter
E-boxes 1-3
SV40 polyA
pEcad-Luc/Rluc
9574 bps
Luciferase (firefly)
f1 ori
Amp^R
ColE1
SV40 polyA
C
2^-ddCt
24h
48h
vehicle
PROAM02-0008
PROAM02-0118
PROAM02-0258
PROAM02-0269

Figure S1: Schematic presentation of the double reporter construct pEcad-luc/Rluc for the high throughput screening (HTS) assay

A) and B) The E-cadherin promoter, containing three critical E-boxes, was cloned upstream of the *Firefly* luciferase gene. The viral HSV-Tk promoter was cloned upstream the *Renilla* luciferase gene. Both promoter-luciferase reporter constructs were cloned in opposite orientation to avoid potential interference of transcription. C) E-cadherin mRNA expression was reduced after treatment of PC-3M-Pro4luc2 cells with 5 μM LMW-compound for 24 and 48 hours. Gene expression is displayed as 2^-ddCt values.

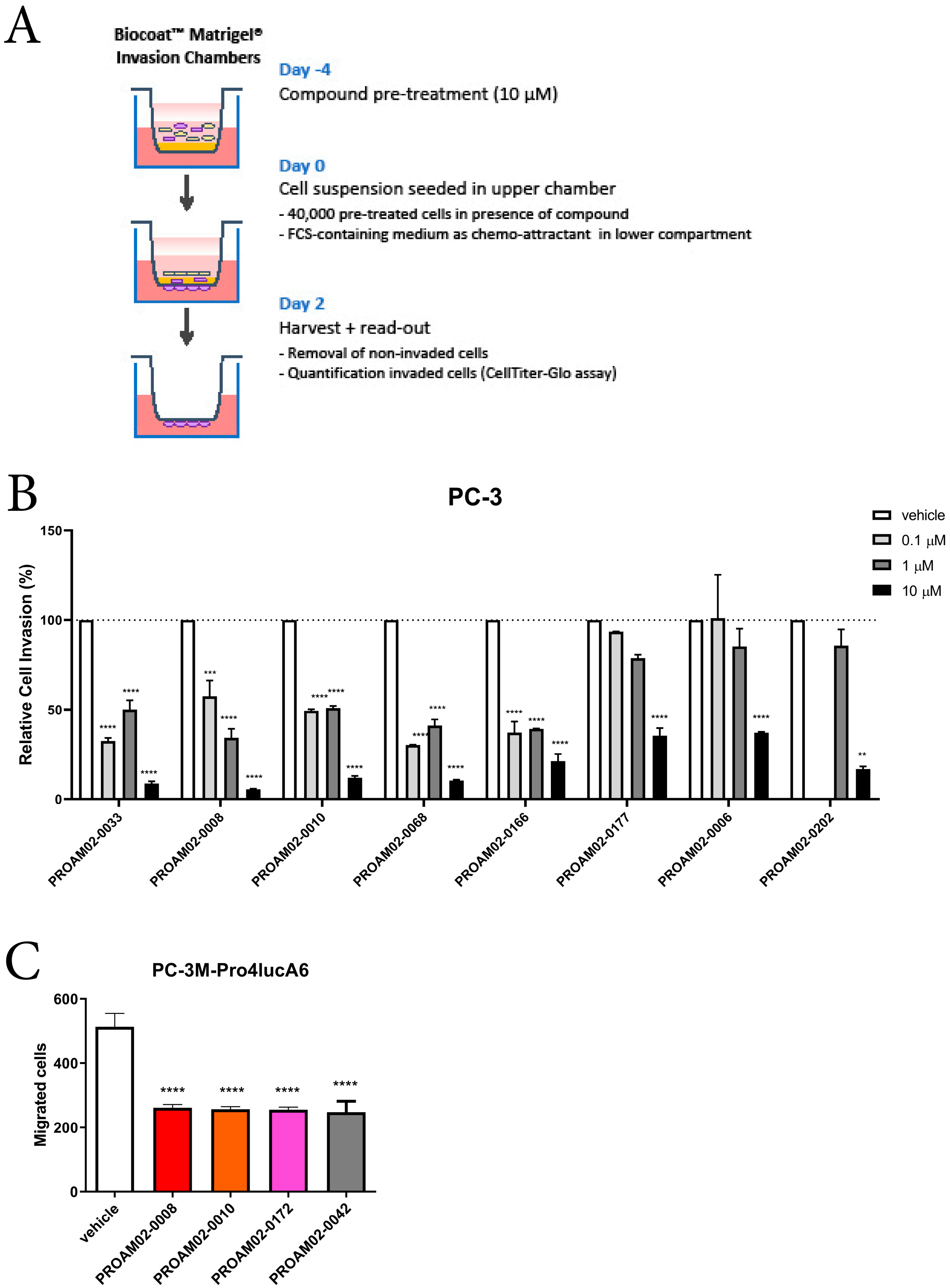
A
Biocoat™ Matrigel® Invasion Chambers
Day -4
Compound pre-treatment (10 µM)
Day 0
Cell suspension seeded in upper chamber
- 40,000 pre-treated cells in presence of compound
- FCS-containing medium as chemo-attractant in lower compartment
Day 2
Harvest + read-out
- Removal of non-invaded cells
- Quantification invaded cells (CellTiter-Glo assay)
B
PC-3
Relative Cell Invasion (%)
150
100
50
0
vehicle
0.1 µM
1 µM
10 µM
PROAM02-0033
PROAM02-0008
PROAM02-0010
PROAM02-0068
PROAM02-0166
PROAM02-0177
PROAM02-0006
PROAM02-0202
C
PC-3M-Pro4lucA6
Migrated cells
600
400
200
0
vehicle
PROAM02-0008
PROAM02-0010
PROAM02-0172
PROAM02-0042

Figure S2: The dose-dependent effect of the LMW-compounds on prostate cancer invasion *in vitro*.

A) Schematic overview of the BioCoat Matrigel invasion assay. B) PC-3 cells were treated with a dose ranging from 0.1 to 10 μM. After 2 days, the effect of the LMW-compounds on invasion was measured. (Relative cell invasion +/-SEM, two-way ANOVA). * $p<0.05$, ** $p<0.01$, *** $p<0.001$ and **** $p<0.0001$ C) The migration of PC-3M-Pro4lucA6 cells was significantly reduced after treatment with 5 μM for 24 hours. Number of migrated cells +/-SEM, one-way ANOVA). **** $p<0.0001$

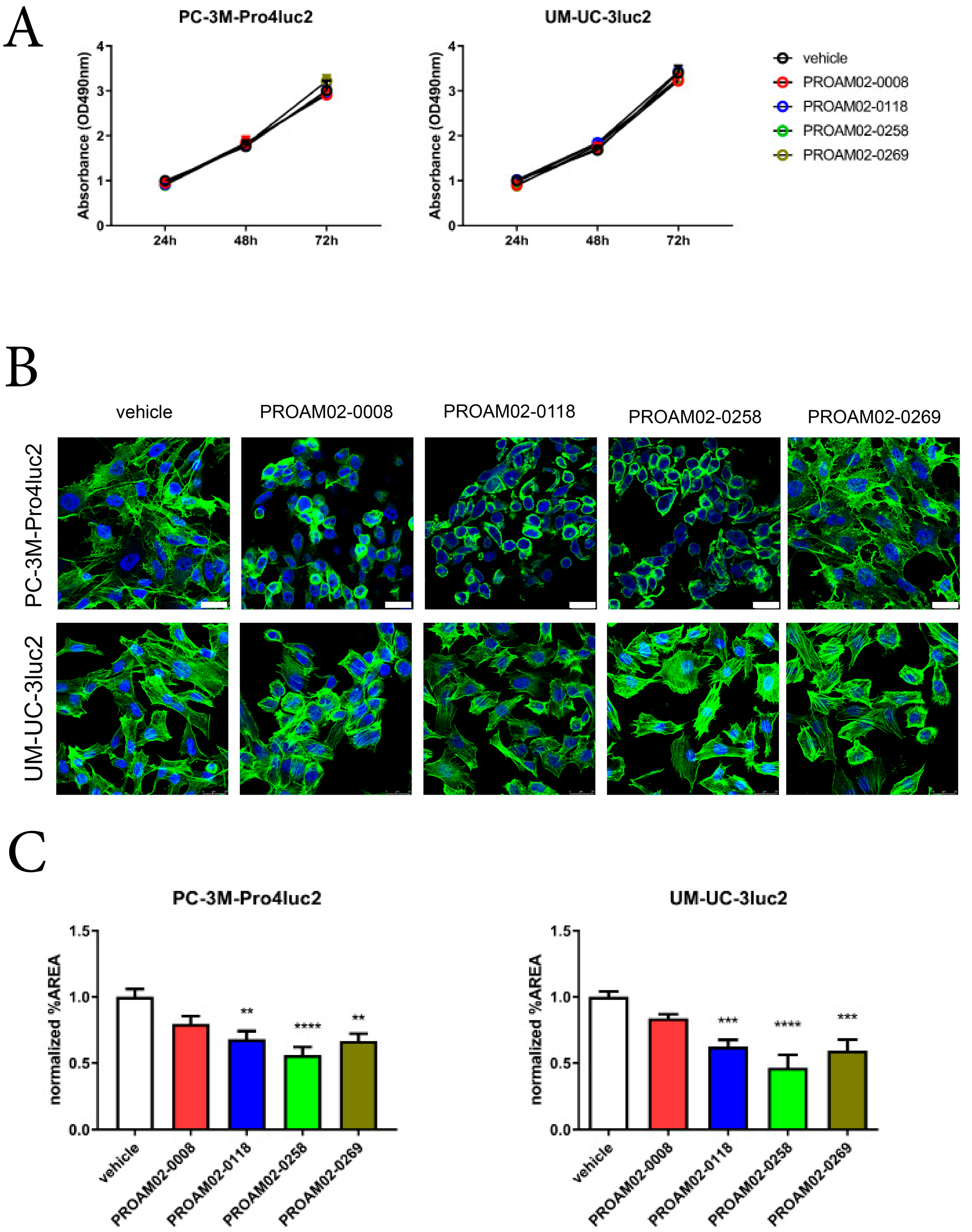

Figure S3: Effects of LMW-compounds on cancer cell proliferation, organization of the actin cytoskeleton and clonogenicity

A) No changes in proliferation were observed after treatment of prostate and bladder cancer cells with 5 μM LMW-compound for 72 hours. (N=3, mean +/- SEM, two-way ANOVA)

B) Treatment of human prostate and bladder cancer cells with selected LMW-compounds for 48 hours induced changes in the actin cytoskeleton indicative of a more sessile, epithelial phenotype. (Phalloidin staining =green, DAPI staining =blue, 63x magnification, scalebar = 25 μm). C) Treatment with 5 μM of LMW-compounds significantly reduced the clonogenic potential of PC-3M-Pro4luc2 and UM-UC-3luc2. Normalized percentage area colonies was quantified by ImageJ. Representative images are displayed below. ** $p<0.01$, *** $p<0.001$ and **** $p<0.0001$ (N=4, mean +/- SEM, one-way ANOVA)

A

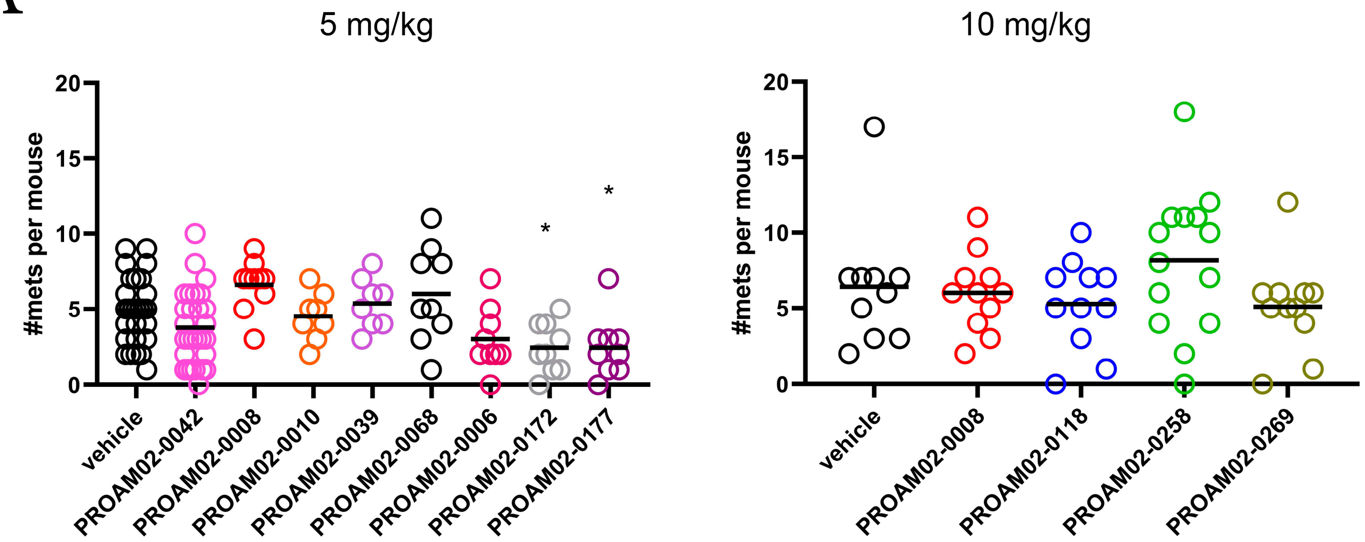

B

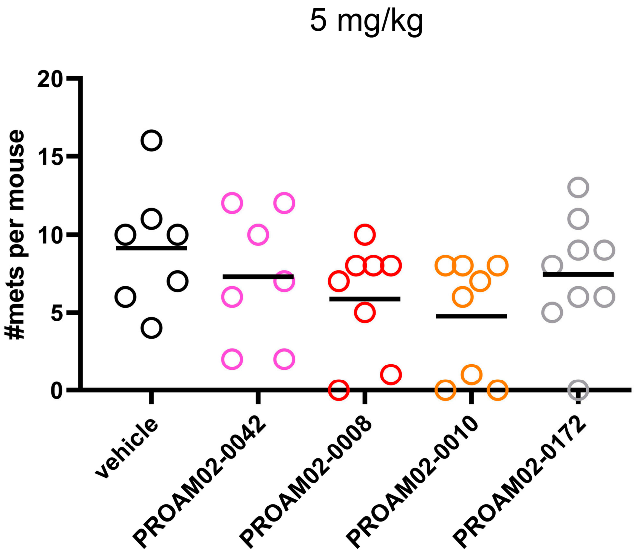

Figure S4: The effect of LMW-compounds on bone metastasis formation and intraosseous tumor growth *in vivo*

A) Screening of the effect of selected LMW-compounds on prostate cancer metastasis formation *in vivo.* Human prostate cancer cells PC-3M-Pro4lucA6 were inoculated into the left cardiac ventricle of male mice and treated by daily intraperitoneal administration of selected LMW compounds. The number of skeletal metastasis were measured. Treatment with 5 mg/kg PROAM02-0172 and -0177 significantly reduced the number of metastases per mouse. (Mean number of metastases per mouse cells, one-way ANOVA). * p<0.05)

B) The effect of the LMW-compounds on the formation of breast cancer metastasis *in vivo*. Human breast cancer cells MDA-MB-231luc were injected in the left cardiac ventricle and treated daily by intraperitoneal injection. The number of skeletal metastasis was scored.

** $P<0.01$, (Mean +/- SEM, two-way ANOVA)

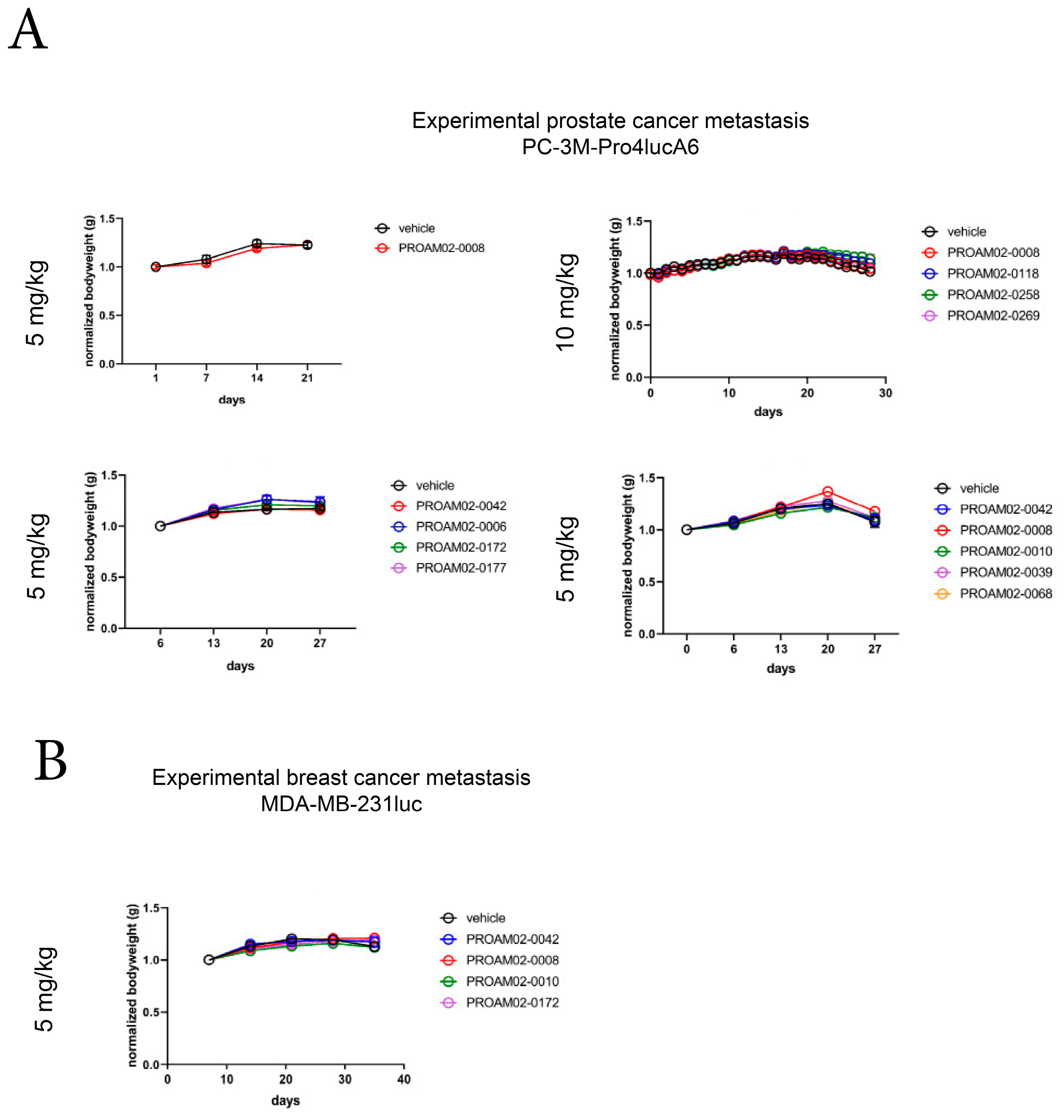

Figure S5: LMW-compound administration does not affect bodyweight

Mice were treated with 5 or 10 mg/kg LMW-compound by daily peritoneal administration. No significant changes in bodyweights were observed.

(Mean +/- SEM, two-way ANOVA)
